# New Challenges in the Study of Cognitive Impairment in Parkinson’s Disease

**DOI:** 10.3390/brainsci16010025

**Published:** 2025-12-25

**Authors:** Sonia Di Tella, Laura Colautti

**Affiliations:** 1 Experimental and Applied Psychology Laboratory, Department of Health and Life Sciences, Università Europea di Roma, 00163 Rome, Italy; sonia.ditella@unier.it; 2 Department of Psychology, Università Cattolica del Sacro Cuore, 20123 Milan, Italy

## 1. Introduction

Parkinson’s disease (PD) ranks as the second most frequent neurodegenerative disorder [1] and is typically recognized by motor difficulties, such as bradykinesia, rigidity, resting tremor, and disturbances with walking and posture [2]. Beyond these hallmark motor signs, individuals may also exhibit non-motor symptoms, including cognitive impairments [3,4].

Cognitive impairment in PD represents one of the most impactful and heterogeneous dimensions of the disorder, shaping autonomy, social functioning, quality of life, and caregiver burden [5,6]. Given the diverse factors involved in cognitive decline [7], this Special Issue integrates perspectives from clinical neuropsychology, neurobiology, and digital innovation to outline advanced, patient-centered methodologies for early diagnosis, monitoring, and treatment of cognitive dysfunctions in PD. This Editorial draws out a multidimensional and integrative perspective on PD-related cognitive impairment, synthesizing the insights offered by the different studies.

A first key point to consider is the increasing role of digital and remote technologies for cognitive evaluation, which represents a crucial component both for improving the accuracy of the assessment and for the early detection and longitudinal monitoring [8]. In this way, Pini and colleagues (contribution 1) led a feasibility study on a telematic version of the Parkinson’s Disease—Cognitive Rating Scale (PD-CRS). It demonstrates that remote cognitive screening is both acceptable and feasible across diverse demographic and clinical profiles. This work directly responds to one of the major challenges in PD cognitive assessment: capturing subtle fluctuations and early cognitive changes with tools that are scalable, accessible, and ecologically valid. Remote assessment platforms also facilitate more frequent evaluations, enabling clinicians to detect changes that may otherwise remain unnoticed in traditional, point-in-time clinical visits. Importantly, such digital methodologies create the conditions for integrating cognitive performance with real-world behavioral, emotional, and lifestyle data.

The transition from digital cognition to neurobiological mechanisms is essential for understanding why cognitive impairment unfolds so heterogeneously in PD. Di Tella and colleagues (contribution 2) carried out a functional connectivity study focusing on large-scale cognitive networks. It shows that the salience network and central executive network support word selection processes in early PD. These results highlight executive dysfunction and suggest that compensatory recruitment of attentionally demanding networks may occur before overt cognitive deficits emerge. Such findings position large-scale functional networks as promising targets for non-invasive interventions and deepen our understanding of the neural architecture underlying early cognitive changes [9].

Complementing functional network evidence, the investigation of structural cholinergic degeneration in the nucleus basalis of Meynert (NBM) reveals a different temporal profile. Sisodia and colleagues (contribution 3) highlight that while NBM atrophy is associated with PD dementia, it does not distinguish individuals with Mild Cognitive Impairment (PD-MCI), nor does it predict short-term postoperative cognitive decline after subthalamic nucleus deep brain stimulation (STN-DBS). This suggests that structural biomarkers may capture later and more advanced stages of degeneration, whereas early cognitive symptoms likely reflect more complex interactions between subtle neural changes, compensatory mechanisms, and psychological factors.

Such interactions are further illuminated by the study on subjective cognitive complaints (SCCs), cognitive reserve (CR), and emotional functioning led by Siri and colleagues (contribution 4). In cognitively unimpaired PD individuals, SCCs were not driven by objective cognitive measures but by anxiety, depression, apathy, and impulsive–compulsive behaviors. At the same time, CR—especially its socially mediated, late-life components—buffered the impact of SCCs on quality of life. This finding not only explains part of the variability observed in clinical trajectories, but also provides a conceptual bridge between neurobiology and treatment responsiveness: individuals with greater reserve or stronger emotional regulation systems may show more effective compensatory functioning even in the presence of subtle neuropathology. Such results widen the evidence showing that CR can represent a protective factor for cognition in PD patients [10,11] as well as for motor functioning [12].

Building on this biopsychosocial foundation, the Special Issue culminates with the theme of cognitive rehabilitation and therapeutic innovation. The systematic review of cognitive training programs conducted by Gattoni and colleagues (contribution 5) demonstrates that multiple rehabilitative modalities—ranging from computer-based and paper-and-pencil approaches to dual-task training, exergaming, and tele-rehabilitation—can improve attention, processing speed, and executive functions in PD. Yet the review also highlights persistent methodological heterogeneity and limited evidence for generalization to everyday functioning. These limitations resonate with the findings from the CR and SCC study: cognitive training effects may depend heavily on individual psychological and contextual factors, including motivation, emotional well-being, and reserve-related compensatory mechanisms. Furthermore, digital tools such as the telematic PD-CRS may offer the infrastructure needed to monitor training effects more continuously and in real-world environments.

Overall, the integrated message emerging from these contributions is that cognitive impairment in PD is an emergent product of interacting biological, psychological, and environmental factors. Structural cholinergic decline may drive later-stage deterioration, but early cognitive symptoms are shaped by network-level functional changes, emotional status, cognitive reserve, and daily context [13]. Digital innovation offers new avenues for early detection and personalized monitoring, while cognitive training provides targeted but variable benefits that depend on an individual’s broader compensatory profile.

Future research should prioritize multimodal, longitudinal designs integrating neuroimaging biomarkers, digital assessments, ecological behavioral data, and clinical and psychosocial profiling to develop predictive models of cognitive decline (e.g., [14]). Cognitive reserve should be systematically incorporated into risk stratification frameworks, and cognitive rehabilitation must be standardized with emphasis on functional outcomes. Finally, hybrid models of in-person and remote neuropsychological assessment will be crucial to ensure both accessibility and validity.

## 2. Conclusions

The studies published in this Special Issue collectively outline a coherent and forward-looking agenda for the field. By interweaving digital innovation, neurobiological insights, psychological determinants, and rehabilitation approaches, they advocate for an integrated, patient-centered strategy to understand and address cognitive impairment in PD. Such a multidimensional vision is essential for developing interventions that are scientifically rigorous, personalized, and meaningful for daily life.

## Abbreviations

The following abbreviations are used in this manuscript:

CR	cognitive reserve
MCI	Mild Cognitive Impairment
NBM	nucleus basalis of Meynert
PD	Parkinson’s disease
PD—CRS	Parkinson’s Disease—Cognitive Rating Scale
SCCs	subjective cognitive complaints
STN-DBS	subthalamic nucleus deep brain stimulation
